# Efficacy and tolerability of preservative-free 0.0015% tafluprost in glaucoma patients: a prospective crossover study

**DOI:** 10.1186/s12886-017-0453-z

**Published:** 2017-04-28

**Authors:** Wonseok Lee, Sunghoon Lee, HyoungWon Bae, Chan Yun Kim, Gong Je Seong

**Affiliations:** 1 Department of Ophthalmology, International St. Mary’s Hospital, Catholic Kwandong University College of Medicine, Incheon, Republic of Korea; 2 Institute of Vision Research, Department of Ophthalmology, Yonsei University College of Medicine, #211Eonjuro, Gangnam-gu, Seoul, 06273 Republic of Korea

**Keywords:** Tafluprost, Preservative, Efficacy, Tolerability

## Abstract

**Background:**

The aim of this work is to evaluate efficacy and tolerability of preservative containing 0.0015% tafluprost and preservative-free 0.0015% tafluprost using a prospective crossover study.

**Methods:**

Primary open angle glaucoma (POAG) and normotensive glaucoma (NTG) patients were randomized enrolled. Group 1 (“NPT to PT”) patients used preservative-free 0.0015% tafluprost (NPT) for 6 months and then changed to preservative containing 0.0015% tafluprost(PT) for 6 months. Group 2 (“PT to NPT”) patients used preservative containing 0.0015% tafluprost for 6 months and changed to preservative-free 0.0015% tafluprost for 6 months. At 1, 3, 6, 7, 9, and 12 months, we measured intraocular pressure for efficacy and graded corneal erosion, tear break-up time (TBUT), and subjective discomfort.

**Results:**

A total of 20 patients and 20 eyes were enrolled. In Group 1 and 2, intraocular pressure was well controlled to approximately 14 mmHg (9.38–18.46% decrease). Generally, subjective satisfaction was improved after changing from PT to NPT (*p* = 0.03) and TBUT using PT was numerically inferior to that using NPT (*p* = 0.06) but not when changing from NPT to PT.

**Conclusion:**

Both preservative containing and preservative-free 0.0015% tafluprost reduced intraocular pressure significantly. In addition, changing medication from PT to NPT might improve subjective satisfaction and tear break up time.

**Trial registration:**

The trial registration number is NCT 03104621 (Apr/1/2017). Retrospectively registered.

## Background

Glaucoma is the leading cause of irreversible visual loss accompanying optic neuropathy and specific visual field deficits. Many factors contribute to the development of glaucoma; however, intraocular pressure (IOP) is the most important factor. The control of IOP is currently the only management option for glaucoma [1–3].

Many anti-glaucoma drugs are delivered via eyedrops, but the effects of lowering IOP vary from 10 to 30% [1–3]. Glaucoma patients typically use anti-glaucoma eyedrops for a long time. Not only the effect of anti-glaucoma eye drops, but also their tolerability are very important to patients.

Among anti-glaucoma eyedrops, prostaglandin analogues are one of the most effective drugs for lowering IOP. The prostaglandin analogue acts by increasing aqueous drainage through the non-conventional uveoscleral pathway [1]. The IOP lowering effect of prostaglandin analogues is approximately 27–35%. Prostaglandin analogues have feweradverse effects compared to other IOP lowering drugs [2, 4]. In this study, we will discuss Tafluprost.

In the process of making anti-glaucoma drugs, preservatives are used widely for preventing denaturation of the eyedrops and preserving the drug for a long time. Benzalkonium chloride (BAK) is the most used preservative and is excellent for safety and stability of drug. However, it causes dry eye, corneal oedema, corneal erosion, and corneal toxicities, thus lowering the long-term tolerability for patients [5–7]. A critical component when managing glaucoma patients is ensuring compliance. However, adverse effects decrease the compliance of regular eye drop application in glaucoma patients.

Once the toxicities of BAK were reported, efforts began to reduce preservatives while preventing denaturation of the drugs. Substitutions for BAK have been found [8–10] and preservative-free disposable packs for one-timeuse have been developed [11].

The adverse effects of prostaglandins are conjunctival injection and pigmentation of both the iris and periocular area [2]. BAK also has toxicities on cornea erosion and dry eye [5–7]. In this study, we investigated the effect of 0.0015% Tafluprost and the tolerability between BAK containing product (Taflotan®) and non-preservative disposable pack product (Taflotan-S®). Many ophthalmologists want to prescribe prostaglandin analogues for effective IOP reduction and non-preservative medications for lowering adverse effects. In those aspects, Taflotan-S® is currently the only glaucoma medication that is commercially available as a non-preservative prostaglandin analogue.

## Methods

This prospective, crossover study protocol followed the tenets of the Declaration of Helsinki and was approved from the Institutional Review Board of Gangnam Severance Hospital, Yonsei University College of Medicine. All subjects were provided informed consent. Between Jan 2013 and Sep 2015, patients from the Gangnam Severance Hospital eye center (Seoul, South Korea) were recruited. Study subjects were the initially diagnosed glaucoma patients. We decided sample size by program (G power®, copyright by Heinrich-Heine-Universitat Dusseldorf), which provides appropriate sample size calculating. With one tailed matched pairs Wilcoxon signed-rank test, we could gain twenty subjects of total sample sizes. Effect size was 80% and α error was 0.05. We constructed cross over study, one group was consisted 10 subjects. This prospective, comparative, crossover study recruited patients into two groups. Group 1 is “NPT to PT”. For the first 6 months, the subjects of group 1 used non-preservative disposable 0.0015% tafluprost product (Taflotan-S®, Santen Pharmaceutical Co. Ltd, Osaka, Japan) and then changed to 0.001% Benzalkonium chloride (BAK), 0.0015% tafluprost product (Taflotan®, Santen Pharmaceutical Co. Ltd, Osaka, Japan) for 6 months. Group 2 is “PT to NPT”. These subjects used Taflotan® for the first 6 months and then changed to Taflotan-S® for 6 months. Subjects used one drop tafluprost (whether preservatives or preservative free) once a day at night. Subjects were randomly arranged into group 1 or group 2. Subjects were randomized by simple randomization. Before subjects were enrolled, the sequence of enrollment was formed, whether preservative product use first or not (group 1or 2). For example, we had the sequence of enrollment by flipping coin; (group) 1-2-1-1-2-1-2-2-2-1-2-1-2-1-2-1-2-2-1-1. If one patient decided to be enrolled at outpatient clinic on fourth subject, (s)he was enrolled to group 1. During the study, subjects stopped tafluprost if severe adverse effects appeared.

Primary open angle glaucoma and normotensive glaucoma patients who came to the outpatient clinic for regular glaucoma check-ups were enrolled. Glaucoma was defined as the patients who had open angle confirmed by gonioscopy, optic nerve cupping (a vertical cup-disc ratio of >0.6) and or notching of the neuroretinal rim and or retinal nerve fiber defects characteristics of glaucoma, and visual field defect (i.e., a glaucoma hemi-filed test result outside normal limits, a pattern standard deviation probability of <5%, or a cluster of three or more non-edge points in location typical of glaucoma, all of which were depressed on a pattern deviation plot at a P level of <5%, and at least one of which was depressed at a P level of <1% on two consecutive visual field tests). Normal tension glaucoma included criteria: repeated measurements of untreated IOP values of < 21 mmHg. Primary open angle glaucoma included criteria: repeated measurements of untreated IOP values of ≥ 22 mmHg. Phakic and pseudophakic eyes were enrolled but aphakic eyes were excluded. Also, eyes that had been taken vitrectomy, trabeculectomy, or through other surgery influencing IOP were excluded. If both eye were glaucoma, the eye which had more severe visual field defect and higher IOP was enrolled. At baseline, we performed visual acuity, IOP, visual field test (SITA-Standard 30–2 programme; HFA II-750i, Carl Zeiss Meditec®, CA, USA), slit lamp examination, and administered a questionnaire.

**Fig. 1:**
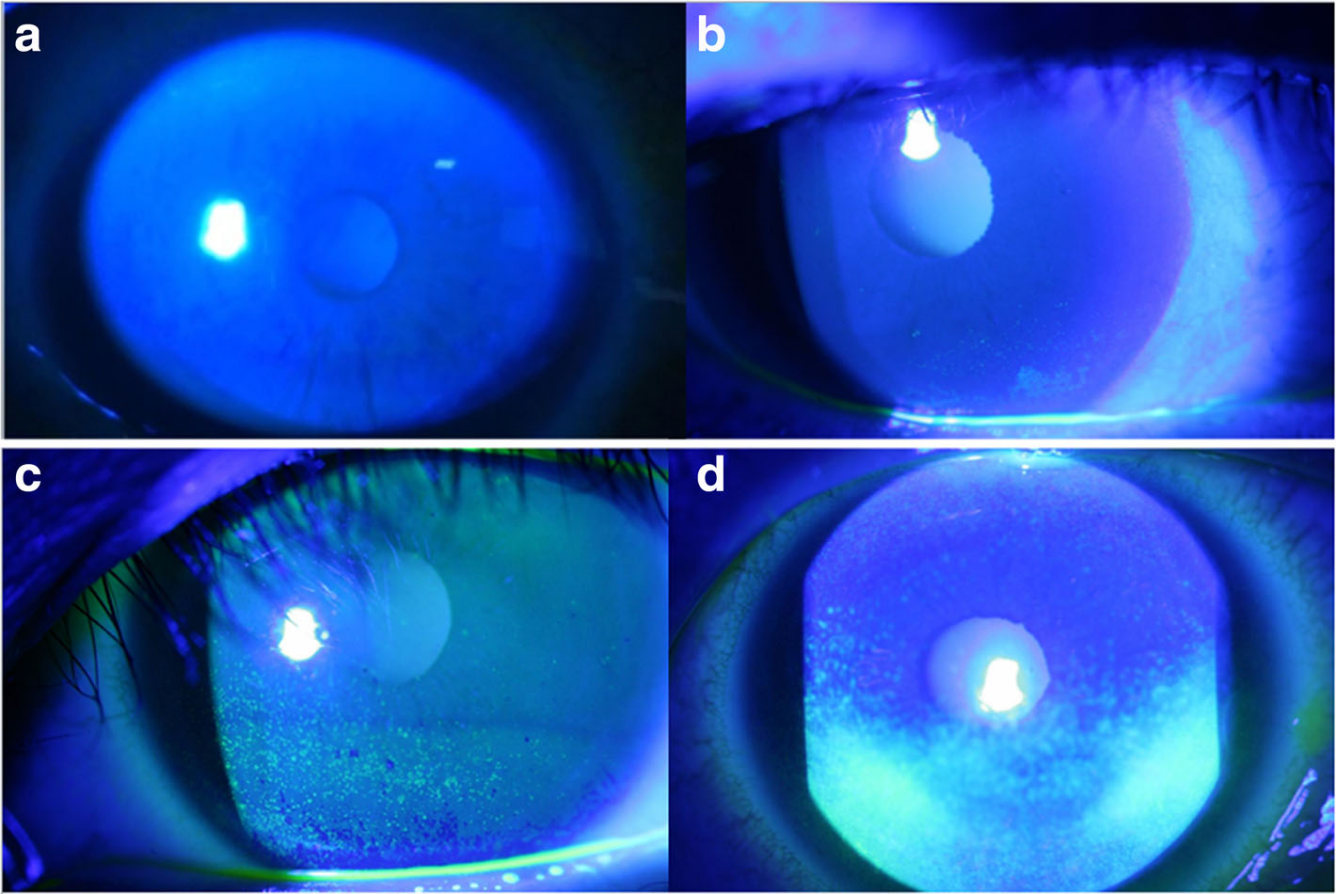
(**a**) Corneal erosion grade 0; Little to no erosion (**b**) Corneal erosion grade 1; erosion on lower than 1/3 of the area of the entire cornea (**c**) Corneal erosion grade 2; erosion on 1/3 to 2/3 of the area of the entire cornea (**d**) Corneal erosion grade 3; erosion on the nearlyentire cornea

Patients were equally randomized in double blind manner to preservative or non-preservative tafluprost. The 20 enrolled patients did not have washout periods between the two products (Taflotan® and Taflotan-S®). Investigators, patients and other study participants were blinded to treatment assignment throughout the study. Evaluator of IOP was also masked to treatment assignment. We checked corneal erosion status, tear break up time, Schirmer test under topical anaesthesia (5% Proparacaine HCl, Alcaine®, Alcon Laboratories Inc., TX, USA), and administered a questionnaire for subjective discomfort after 1, 3, and 6 months using one drug. After changing to the other product, similar follow up tests occurred at 1, 3, and 6 months. IOP was checked by Goldmann applanation tonometry in the morning during the hours of clinic operation (9:00 am to 12:00 pm). We modified Ocular surface disease Index (OSDI); questionnaire questions included subjective discomfort such as stinging sensation, itching, dryness, foreign body sensation, and conjunctival injection. The subjective discomfort scaleincluded “0” (no discomfort), “1” (mild discomfort), and “2” (severe discomfort) [3]. The scores of each question were summed. Corneal erosion scales were scored according to the area of erosion. Little to no erosion was “0”, erosion on 1/3 of the area of the entire cornea was “1”, erosion on 2/3 of the area of the entire cornea was “2”, and erosion on the entire cornea was “3” (Fig. 1) [7]. For tear secretion, schirmer test paper was placed into the conjunctival sac at the point of 1/3 from lateral canthus under topical anaesthesia (5% Proparacaine HCl, Alcaine®, Alcon Laboratories Inc., TX, USA). After 5 min, we checked the wet height with tear (mm). Tear breakup time was checked by slit lamp exam under corneal fluorescein dye. We asked patients not to blink, and the time was counted until tear film was torn apart (seconds).

All data were analysed by SPSS® (IBM®, SPSS Statistics ver. 20) using student *t*-test, Chi square test and Wilcoxon signed rank test. In case of multiple comparisons, *t*-test and Chi-square tests with Bonferroni correction were used.

## Results

### Subject characteristics

Among the 20 subjects, 10 were assigned to each group. The mean age was 55.26 ± 14.22 years. The mean age of group 1 was 55.90 ± 15.98 years and group 2 was 54.55 ± 12.90 years. Five males and five females were in each group. Eleven subjects had systemic disease (two had diabetes mellitus and nine had hypertension). Three subjects smoked (Table 1). Two patients were primary open angle glaucoma patients and 18 were normotensive glaucoma patients. The mean visual acuity (LogMAR) was 0.116 in group 1 and 0.096 in group 2. The mean deviation (MD) of the Humphrey test was −7.50 ± 6.43 dB and the visual field index (VFI) was 78.31 ± 22.77%. MD and VFI of group 1 were −8.56 ± 7.66 dB and 73.20 ± 26.35%. MD and VFI of group 2 were −6.32 ± 4.90 dB and 84.00 ± 17.79% (Table 2).

**Table 1:** Patients demographics

	Total (*n* = 20)	Group 1 (*n* = 10)	Group 2 (*n* = 10)	*p*
Age (years)	55.26 ± 14.22^a^	55.90 ± 15.98^a^	54.55 ± 12.90^a^	0.73
Sex (M:F)	10:10	5:5	5:5	1.00^†^
Systemic disease (DM)	2	1	1	1.00^†^
Systemic disease (Hypertension)	9	5	4	0.52^†^
Smoking history	3	2	1	0.06^†^
Laterality (R:L)	8:12	5:5	3:7	0.20^†^

*DM* diabetes mellitus

^a^average ± standard deviation; *p*, *p*-values; ^†^chi-square test

**Table 2:** Ophthalmic characteristics

	Total (*n* = 20)	Group 1 (*n* = 10)	Group 2 (*n* = 10)	*p*
POAG:NTG	2:18	1:9	1:9	1.00^†^
Visual acuity (LogMAR)	0.106	0.116	0.096	0.98
IOP (mmHg)	16.84 ± 2.75^a^	16.70 ± 3.02^a^	17.00 ± 2.59^a^	0.74
MD (dB)	−7.50 ± 6.43^a^	−8.56 ± 7.66^a^	−6.32 ± 4.90^a^	0.32
VFI (%)	78.31 ± 22.77^a^	73.20 ± 26.35^a^	84.00 ± 17.79^a^	0.31

*POAG* primary open angle glaucoma, *NTG* normotensive glaucoma, *IOP* intraocular pressure, *MD* mean deviation, *VFI* visual field index

^a^average ± standard deviation; *p*, *p*-values; ^†^chi-square test

### Intraocular pressure reduction

**Fig. 2:**
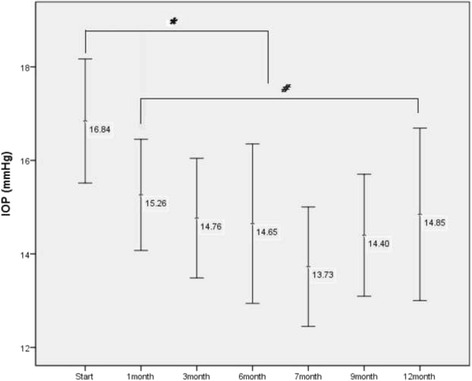
Intraocular pressure (IOP) before and after using 0.0015% tafluprost. Indicated numbers are the means of intraocular pressure before using tafluprost and after 1, 3, 6, 7, 9, and 12 months of use. 1 month after using tafluprost, IOP was well maintained for 12 month. (* : *p* < 0.001, # : *p* > 0.05)

IOP was analysed for the 20 patients. Both products contained 0.0015% tafluprost, but differed in the BAK content. To analyse the IOP reduction effect of tafluprost, IOP was checked before and after using tafluprost for 1, 3, 6, 7, 9, and 12 months. The mean IOP before using tafluprost decreased from 16.84 ± 2.75 mmHg to 14.85 ± 3.05 mmHg (11.81%) after 12 months of use. The means of IOP at 1, 3, 6, 7, 9, and 12 months were significantly lower compared to the IOP before using tafluprost (*p* < 0.01) (Table 2, Fig. 2).

### Subjective discomfort

**Fig. 3:**
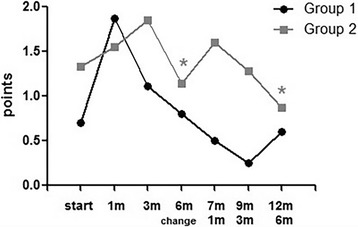
Subjective discomfort in Group 1 (NPT → PT) and Group 2 (PT → NPT). Before exchanging product (6 m) and after 6 month (12 m), the reduction of score was significant in Group 2. (*p* = 0.03)

Subjective discomfort (summed score of stinging sensation, itching, dryness, foreign body sensation, and conjunctival injection) was significantly improved in group 2 (PT to NPT). Before exchanging products, the scores of subjective symptoms was 1.14 ± 0.69 (points). After 6 months of NPT (at 12 months), the scores of subjective symptoms were significantly improved to 0.87 ± 1.72 (points) (*p* = 0.03) (Fig. 3, Table 3).

**Table 3:** The means of subjective discomfort grading, corneal erosion grading, Schirmer test and tear break up time (TBUT) between Group 1 and 2

	Group 1 (Taflotan-S® → Taflotan®)							Group 2 (Taflotan® → Taflotan-S®)						
	start	1 month	3 month	6 month	7 month	9 month	12 month	start	1 month	3 month	6 month	7 month	9 month	12 month
Subjective discomfort (points)	0.70 ± 0.67	1.87 ± 1.24 *p* = 0.02*	1.11 ± 1.16 *p* = 0.31*	0.80 ± 1.39 *p* = 0.91*	0.50 ± 0.83 *p* > 0.99* *p* = 0.31^†^	0.25 ± 0.46 *p* > 0.99* *p* = 0.10^†^	0.60 ± 0.89 *p* = 0.78* *p* > 0.99^†^	1.33 ± 1.00	1.55 ± 1.66 *p* = 0.34*	1.85 ± 1.95 *p* = 0.26*	1.14 ± 0.69 *p* = 0.24*	1.60 ± 2.07 *p* = 0.10* *p* = 0.56^†^	1.28 ± 1.38 *p* = 0.11* *p* = 0.65^†^	0.87 ± 1.72 *p* = 0.08* *p* = 0.03^†^
Cornea erosion (points)	0.30 ± 0.48	0.25 ± 0.46 *p* = 0.56*	0.22 ± 0.44 *p* = 0.56*	0.40 ± 0.51 *p* = 0.56*	0.50 ± 0.54 *p* = 0.56* *p* > 0.99^†^	0.50 ± 0.53 *p* = 0.31* *p* = 0.65^†^	0.60 ± 0.54 *p* = 0.31* *p* = 0.56^†^	0.55 ± 0.52	0.33 ± 0.50 *p* = 0.15*	0.28 ± 0.48 *p* = 0.08*	0.14 ± 0.37 *p* = 0.15*	0.80 ± 0.83 p > 0.99* *p* = 0.15^†^	0.42 ± 0.53 *p* = 0.56 *p* > 0.99^†^	0.25 ± 0.46 *p* = 0.18* *p* > 0.99^†^
Schirmer (mm)	5.80 ± 3.88	2.62 ± 2.87 *p* = 0.20*	4.22 ± 3.15 *p* = 0.34*	4.60 ± 3.97 *p* = 0.40*	4.83 ± 6.27 *p* = 0.34* *p* = 0.58^†^	5.37 ± 6.04 *p* = 0.93* *p* = 0.68^†^	4.60 ± 4.56 *p* = 0.34* *p* = 0.78^†^	3.33 ± 3.04	5.66 ± 7.01 *p* = 0.88*	5.28 ± 3.40 *p* = 0.22*	5.14 ± 3.67 *p* = 0.40*	4.40 ± 1.34 *p* = 0.46* *p* = 0.70^†^	6.85 ± 5.04 *p* = 0.10* *p* = 0.25^†^	5.00 ± 3.02 *p* = 0.23* *p* = 0.73^†^
TBUT (sec)	5.80 ± 2.39	3.25 ± 1.28 *p* = 0.03*	4.33 ± 1.93 *p* = 0.23*	5.00 ± 1.88 *p* = 0.32*	4.83 ± 1.16 *p* = 0.28* *p* = 0.71^†^	5.00 ± 2.72 *p* = 0.73* *p* = 0.93^†^	3.60 ± 2.07 *p* = 0.06* *p* = 0.85^†^	4.55 ± 2.18	5.44 ± 2.29 *p* = 0.16*	5.00 ± 2.23 *p* = 0.14*	4.42 ± 1.71 *p* = 0.71*	3.60 ± 0.89 *p* = 0.15* *p* = 0.31^†^	4.14 ± 1.06 *p* = 0.67* *p* = 0.48^†^	4.75 ± 1.83 *p* = 0.60* *p* = 0.52^†^

*comparisons with before using tafloprost (start)

^†^comparisions with the 6 months, in other words, before changing medication

In group 1 (NPT to PT) after 1 month using NPT, subjective symptoms were significantly worsened. At baseline, the score of subjective symptoms was 0.70 ± 0.67 (points) and after 1 month using NPT, the score increased to 1.87 ± 1.24 (points) (*p* = 0.02). This may be due to conjunctival redness, which is the most common adverse effect of prostaglandin analogues. After 6 months, the subjective symptoms score recovered to 0.80 ± 1.39 (points) (Fig. 3, Table 3). After exchanging medication to PT, subjective symptoms did not severely worsen.

### Corneal erosion grade/Schirmer test

**Fig. 4:**
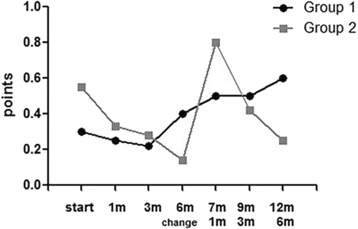
Corneal erosion grade in Group 1 (NPT → PT) and Group 2 (PT → NPT). Corneal erosion was increased using PT, but it was not statistically significant

In group 1, using NPT and changing to PT increased corneal erosion grade, in other words, worsened corneal erosion. Before medication, the score of corneal erosion was 0.30 ± 0.48 (points) but after 12 months, the score increased to 0.60 ± 0.54 (points). PT might make corneal erosion more severe, but it was not statistically significant. In group 2, using NPT increased corneal erosion from 0.14 ± 0.37 to 0.80 ± 0.83, but it was not statistically significant (Fig. 4, Table 3).

**Fig. 5:**
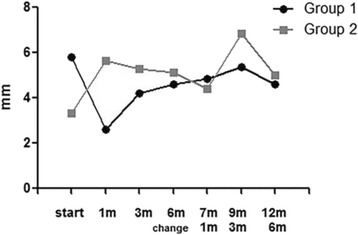
The results of Schirmer test in Group 1 (NPT → PT) and Group 2 (PT → NPT)

Throughout the study, the Schirmer test results were not changed significantly by changing medication (Fig. 5, Table 3).

### Tear break up time (TBUT)

**Fig. 6:**
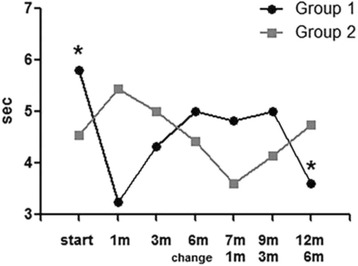
The results of tear break up time (TBUT) in Group 1 (NPT → PT) and Group 2 (PT → NPT). Comparison between baseline TBUT and after 12 months of treatment showed a slightly decreased TBUT in Group 1 (NPT → PT). (*p* = 0.06)

In group 1 (NPT to PT) after 1 month of using NPT, tear breakup time (TBUT) was significantly worsened. At baseline, the TBUT score was 5.80 ± 2.39 (sec), but after 1 month using NPT, the TBUT score decreased to 3.25 ± 1.28 (sec) (*p* = 0.03). However, after 6 months, the scores recovered to the baseline score, 5.00 ± 1.88 (sec) (Fig. 6, Table 3). PT made tear break up time worse. In group 1 before exchanging to PT (at 6 months), tear break up time was 5.00 ± 1.88 (sec). After using PT (at 12 months), tear break up time was decreased to 3.60 ± 2.07 (sec) (*p* = 0.06) (Fig. 6, Table 3).

## Discussion

0.0015% Tafluprost is effective in lowering intraocular pressure (IOP) in many studies and is comparable to other prostaglandin analogues (Latanoprost, Travoprost, Bimatoprost). According to Kuwayama et al., after applying Tafluprost for 2 months, IOPs were reduced by 4.3 ± 5.2 mmHg in primary open angle glaucoma patients, 2.4 ± 2.5 mmHg in normotensive glaucoma patients, and 5.6 ± 7.1 mmHg in angle closure glaucoma patients [1]. Hwang et al. showed that 0.0015% Tafluprost reduced IOP of primary open angle glaucoma patients to about 4.6 mmHg (23.0%) in 1 month, 5.1 mmHg (25.5%) in 3 months, and 4.9 mmHg (24.5%) in 6 months [11]. Similarly, our study found that 0.0015% Tafluprost reduced IOP significantly and maintained reduced IOP for one year.

Subjective discomfort was improved after changing from PT to NPT (*p* = 0.03). However, other studies did not say that NPT was superior to PT in subjective discomfort. The product of Tafluprost (Taflotan®) contains a relatively low concentration (0.001%) of BAK compared to other prostaglandin analogue products (Xalatan® from Pfizer: 0.02% BAK; Lumigan® from Allergan: 0.005% BAK; Travatan Z® from Alcon: sofzia). Because of the relatively low concentration of BAK, Taflotan® is thought toinduce fewer ocular surface problems and patient discomfort. In this study, the differences of subject discomfort were not definite after exchanging the products (PT to NPT or NPT to PT).

Suzuki et al. evaluated corneal toxicities of anti-glaucoma eye drops in severe superficial punctate keratopathy. Corneal status and tear break up time were checked for 12 weeks using 0.0015% Tafluprost (0.001% BAK), 0.005% Latanoprost (0.02% BAK), 0.004% TravoprostZ (sofzia), and 0.03% Bimatoprost (0.005% BAK). This study reported that BAC concentrations under 0.003% causeda few corneal toxicities, but the ones above 0.005% BAK aggravated corneal erosion and shortened tear break up time [7]. Pinheiro et al. found that 0.02% BAK aggravated corneal abrasions from 14.65 mm^2^ to 66.57 mm^2^ to87.26 mm^2^ in area. Iatrogenic corneal abrasions were healed completely and recovered corneal stroma (Descemet’s membrane) structure in the Tafuprost use group, but delayed corneal healing markedly in the Latanoprost use group [5]. Fogagnolo et al. studied the confocal microscopic findings of non-preservative tafluprost and latanoprost in glaucoma patients for 1 year. Clinical effects (IOP reduction) was similar in both group, 3.6–4.2 mmHg. However, corneal nerve branching patterns were significantly different between both groups. None of the patients without beading at baseline developed beading at the end of the study in non-preservative tafluprost group, whereas this occurred in 6/8 (75%) patients treated with latanoprost [12].

In summary, a low concentration of BAK (under 0.003%) causes few corneal toxicities and Tafluprost has less toxicities compared to other prostaglandin analogues. Thus, 0.001% BAK containing Tafluprost may have little chance to cause corneal toxicities. However, because the non-preservative product improved subjective satisfaction, recommending non-preservative product to glaucoma patients is beneficial for maintaining anti-glaucoma eye drop compliance for a long period.

In group 1, even with the non-preservative product, subjective discomfort increased significantly after 1 month. We think that conjunctival injection mostly influenced this situation. The most common adverse effect of prostaglandin analogues is conjunctival injection. Conjunctival injections are most severe during the first 2 weeks, and gradually subside after that. In the same manner, patients in our study were relieved from conjunctival injection after 3 months.

Our study population had many normotensive glaucoma (NTG) patients, one primary open angle glaucoma (IOP over 21 mmHg), and nine normotensive glaucoma (IOP under 21 mmHg) patients. In NTG patients, IOP reduction following prostaglandin analogue use commonly ranged from 15.3% to 22.6%, even though there were non-responders to prostaglandin analogues (0–15%) [13, 14]. It is thought that the results in this study (9.38–18.46% IOP reduction) showed ordinary efficacy.

This study had some limitations. First, the number of enrolled subjects for this study was small. Second, this study was designed for only one year. To evaluate the effect and tolerability of the drug, more subjects and a longer follow-up period are needed. Third, we did not have washout periods. Commonly, crossover studies for drugs have clearance periods for the initial drug to be cleared from the system before starting the second drug. However, maintaining anti-glaucoma drug treatment is important for glaucoma patients, thus making a long wash out period unfeasible.

This study is the first prospective designed crossover study for evaluating efficacy and tolerability of 0.0015% Tafluprost. A crossover study is a randomized, controlled longitudinal study in which subjects receive a sequence of different treatment. Nearly all crossover study designs have balance, which means that all subjects should receive the same number of treatments and that all subjects participate for the same number of periods. A crossover study has advantages in the reduced confounding covariates. Each crossover patient serves as his or her own control, so crossover study design can reduce confounding covariates. We performed our study for two different drugs in one subject; thereby many variables could be controlled.

Crossover studies do have some limitations. First is the order effect. Treatment order may affect the outcome. An example might be a drug with many adverse effects given first, making patients taking a second, less harmful medicine more sensitive to any adverse effect. Second is the learning effect. Patients learn how to better apply the drug over time. Therefore, the second drug may have been more carefully applied than the first.

## Conclusion

0.0015% tafluprost reduced intraocular pressure significantly, whether it contained preservative or not. In addition, changing from preservative containing 0.0015% Tafluprost to preservative-free 0.0015% Tafluprost improved subjective satisfaction and tear break up time. Preservative free Tafluprost may give glaucoma patients not only treatment effect but also improve satisfaction.

## Abbreviations

BAK: Benzalkonium chloride
IOP: Intraocular pressure
NPT: Non-preservative tafluprost
NTG: Normal-tension glaucoma
POAG: Primary open angle glaucoma
PT: Preservative contained tafluprost
TBUT: Tear break up time
